# Portable Instrument for Hemoglobin Determination Using Room-Temperature Phosphorescent Carbon Dots

**DOI:** 10.3390/nano10050825

**Published:** 2020-04-26

**Authors:** Fabio Murru, Francisco J. Romero, Roberto Sánchez-Mudarra, Francisco J. García Ruiz, Diego P. Morales, Luis Fermín Capitán-Vallvey, Alfonso Salinas-Castillo

**Affiliations:** 1 Department of Analytical Chemistry, Faculty of Sciences, University of Granada, 18071 Granada, Spain; 2 Department of Electronics and Computer Technology, Faculty of Sciences, University of Granada, 18071 Granada, Spain; 3 ECsens Group, University of Granada, 18071 Granada, Spain; 4 Unit of Excellence in Chemistry Applied to Biomedicine and the Environment, University of Granada, 18071 Granada, Spain

**Keywords:** carbon dots, hemoglobin determination, luminescence, room temperature phosphorescence, portable instrumentation

## Abstract

A portable reconfigurable platform for hemoglobin determination based on inner filter quenching of room-temperature phosphorescent carbon dots (CDs) in the presence of H_2_O_2_ is described. The electronic setup consists of a light-emitting diode (LED) as the carbon dot optical exciter and a photodiode as a light-to-current converter integrated in the same instrument. The reconfigurable feature provides adaptability to use the platform as an analytical probe for CDs coming from different batches with some variations in luminescence characteristics. The variables of the reaction were optimized, such as pH, concentration of reagents, and response time; as well as the variables of the portable device, such as LED voltage, photodiode sensitivity, and adjustment of the measuring range by a reconfigurable electronic system. The portable device allowed the determination of hemoglobin with good sensitivity, with a detection limit of 6.2 nM and range up to 125 nM.

## 1. Introduction

In recent years, optical chemical sensing has been a growing research area in many scientific fields as an alternative to expensive and complex conventional analytical procedures [1]. These sensors are based on the monitoring of different optical parameters to obtain the analyte information, such as absorption [2], luminescence intensity [3], luminescence lifetime [3,4], or refractive index [5]. Luminescence-based sensors are highly interesting due to their sensitivity and simplicity, sometimes in combination with smartphones, resulting in portable devices [6,7]. In addition, detection by phosphorescence at room temperature (RTP) offers several advantages over fluorescence, including improved selectivity, a lower emission lifetime, and elimination of spectral interferences from light scattering or autofluorescence.

The present study focuses on the use of the intensity and lifetime of RTP, which allows a sensitive, fast, and reliable determination of the analyte concentration [8,9]. Different methods exist to obtain the decay rate of the excited state, which is a measure of luminescence lifetime, some based on frequency domain analyses, which require costly instrumentation or complex signal processing steps, such as that proposed by Franke et al. [10] or Chen et al. [11]. Others follow direct time-domain techniques, which, in most cases, require high-speed complex readout circuits due to their short lifetimes [12,13]. However, these solutions disrupt the current trend of wireless chemical sensors (WCS), which, within the Internet of Things (IoT) paradigm, aims to make ubiquitous analytical bio-chemical sensing a reality [14]. This paper presents the development and validation of a WCS that uses room-temperature phosphorescence determination of bio-analytes, hemoglobin in this particular case, based on a windows-based algorithm implemented in a reconfigurable device [15].

Hemoglobin (Hb) is a protein that plays a vital role in transporting molecular oxygen through the blood from the respiratory organs (lungs or gills) to the various parts of the body and, in turn, the main portion of CO_2_ from the different organs of the body to the respiratory organs. Hb is a tetrameric metalloprotein that has a quaternary structure composed of four globular protein subunits, each of which contains a non-protein heme group with an iron atom in the ferrous state chelated to four rings of protoporphyrin. Currently, the measurement of Hb plays a crucial role in identifying diseases such as anemia (low Hb level) and polycythemia (high Hb level). The Hb test is also very important during pregnancy, since these diseases are associated with an increased risk of premature birth. Different procedures have been proposed for Hb determination in clinical diagnosis such as optical [16,17], electrochemical [18], or liquid chromatography [19]. A widely used method is the spectrophotometric procedure based on the Van Kampen–Zijlstra reagent, although it uses the toxic alkaline cyanide as a reagent among other disadvantages [20]. Several carbon dot (CD) fluorescent sensors for Hb determination have been published in recent years [21,22]. Therefore, easy, environmentally friendly, and precise assays for the quantitative analysis of Hb is of interest for clinical and physiological diagnosis.

Among the current luminescent nanoparticles (NPs) used in these kind of applications, novel CDs have attracted the interest of many researchers due to their unique properties such as tunable photoluminescence, wavelength-dependency excitation, good photostability, water solubility, low toxic effects, and biocompatibility [23,24]. These properties, together with the CDs’ ability to interact with analytes causing a luminescence quenching, makes CDs a perfect candidate for analyte and bio-analyte determination [3,15,25]. Recently, the phenomenon of phosphorescence at room temperature has been described in both solutions and solid state for CDs [26].

Nevertheless, the variability in the optical properties in every batch synthesis of CDs is one of the limiting factors, when using them is considered for portable instruments, since it would involve multiple calibration steps, firmware updates, or even hardware changes. To overcome these limitations, we considered the use of reconfigurable electronics, which makes it possible to adapt the WCS to the inherent variability in the optical response of different batch syntheses. The feasibility of this approach has been successfully demonstrated for diverse analog sensing applications, such as temperature monitoring and electrochemiluminescent determination [27,28]. To that end, we present a portable instrument with wireless transmission capability for Hb determination using a time domain analysis to obtain both phosphoresce intensity and the lifetime of a luminescence exponential decay, avoiding the use of fast instrumentation or high-performance electronics components.

## 2. Materials and Methods

### 2.1. Reagents and Materials

All the chemicals used in this study were of analytical quality and were used without further purification. Anhydrous citric acid, sodium hydrogen phosphate, sodium hydroxide, and hydrochloric acid were purchased from Panreac Química SLU (Barcelona, Spain). Sodium dihydrogen phosphate, ethylenediamine, hemoglobin powder, and hydrogen peroxide 30% (*v*/*v*) were purchased from Sigma Aldrich Merck (Madrid, Spain). The phosphate buffers (0.02 M NaH_2_PO_4_/Na_2_HPO_4_ pH 1.5–9.5) were prepared by dissolution of the needed reagents in water, and the pH was adjusted by adding 1 M or 0.2 M HCl and NaOH. The working solutions of H_2_O_2_ and hemoglobin were prepared daily with the dilution of the standards in water. All the aqueous solutions were prepared in purified water (resistance 18.2 MΩ·cm) obtained from a Milli-RO 12 plus Milli-Q station (Millipore, Bedford, MA, USA).

### 2.2. Instrumentation

Microwave MicroSYNTH (Milestone Srl, Sorisole, BG, Italy) was used for CD synthesis. High-resolution transmission electron microscopy (HR-TEM) images were obtained from an FEI TITAN G2 60–300 field-emission instrument (Thermo Scientific™, Waltham, MA, USA) equipped with a HAADF detector. The samples were prepared at room temperature in air by depositing a drop of aqueous solution of CDs on a commercial 400 μm mesh carbon Cu-grid. Fourier transform infrared spectra (FTIR) were obtained using a Spectrum Two FTIR spectrometer (PerkinElmer Inc., Waltham, MA, USA). The X-ray diffraction (XRD) was carried out on a D2 phaser diffractometer (Bruker, Karlsruhe, Germany). X-ray photoelectron spectroscopy (XPS) analyses were done on a Kratos Axis Ultra-DLD (Kratos Analytical, Manchester, UK). All of these studies were performed at the Centre of Scientific Instrumentation (University of Granada, Spain). Dynamic light scattering (DLS) measurements were done on a Zetasizer Nanoseries, Nano-ZS90 (Malvern Panalytical, Malvern, Worsts, UK).

Phosphorescence measurements were obtained with a Cary Eclipse UV–Vis fluorescence spectrophotometer (Varian Iberica, Madrid, Spain) equipped with a xenon discharge lamp (peak power equivalent to 75 kW), Czerny–Turner monochromators, and an R-928 photomultiplier tube, which is red sensitive (900 nm), with manual or automatic voltage controlled by Cary–Eclipse software (Agilent, Santa Clara, CA, USA, Cary OS/2 software) for Windows 95/98/NT systems. For the spectra of RTP, the samples were excited at 340 nm and the emission spectra were measured in a wavelength range of 350 to 650 nm, integration time (*t_g_*) of 5 ms, and delay time (*t_d_*) of 0.2 ms in phosphorescence mode. The photomultiplier voltage was 800 V, and the excitation and emission slits were 10 nm. All measurements were made in a quartz cell with a 10 mm optical path. The UV–Vis spectra were collected using an Agilent 8453 diode array spectrophotometer (Agilent Technologies, Santa Clara, CA, USA). The pH was measured using a Crison micropH 2000 pH meter (Hach Lange Spain, Barcelona, Spain). Finally, a proprietary portable device was used to measure the luminescence attenuation at increasing concentrations of hemoglobin.

### 2.3. Synthesis of the CDs

In this paper we used one-pot synthesis to prepare soluble CDs in water without any surface modification by hydrothermal treatment. An aqueous mixture of 10 g of citric acid and 5 mL of ethylenediamine was heated at 180 °C in a 50 mL Teflon-lined steel autoclave for 8 h. After cooling to room temperature, the resultant yellow solution was centrifuged at 3500 rpm for 10 min. The CDs were purified by dialysis (cut-off 1 kD) for 24 h to remove unreacted materials. The CDs, synthesized as a pale-yellow powder (0.2% yield calculated after lyophilization), were stored at room temperature until their use. A standard solution of 0.24 mg·mL^−1^ CDs was prepared in water with the help of an ultrasonic bath sonicator.

### 2.4. Measuring Setup for the Portable Device

**Figure 1:**
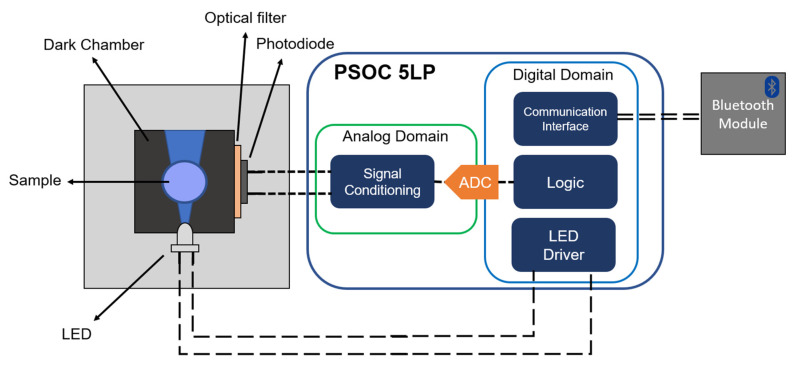
Block diagram of the developed device showing the electronics module and the measurement setup.

The measuring setup basically consisted of three main blocks: an exciting light source, a photodiode, and the reconfigurable analog/digital controller. The UV LED EOLD-365-525 (OSA Opto Light, Berlin, Germany) was used as the excitation source (λ = 365 nm) of the CDs, which were located in a dark chamber specially designed for this purpose. The luminescence emission was acquired using a photodiode S2387-66R (Hamamatsu Photonics K.K., Hamamatsu, Japan) aligned perpendicularly with respect to the emission pattern of the UV LED (as shown in Figure 1). To consider only the effect of the CDs luminescence, an optical filter (KOOD International, Japan) was placed directly in front of the photodiode to avoid the influence of undesirable light reflections.

The control of the excitation LED and the acquisition of the signal from the photodiode were carried out using a programmable system-on-chip (5LP, Cypress Semiconductor, San Jose, CA, USA), specifically the CY8CKIT-010 development kit.

This low-power system-on-chip (SoC) has a reconfigurable architecture that integrates a programmable analog domain together with a powerful signal processing engine (32-bit Arm^®^ Cortex^®^-M0+), which allows both analog signal conditioning and digital processing, and provides a communication control interface.

The block diagram of the developed instrument is schematized in Figure 1. As can be seen, only the photoelectronic module (LED and photodiode) and the Bluetooth module were the out-of-chip components. Thus, the signal obtained from the photodiode was fully conditioned using the analog domain of the PSoC before its conversion to the digital domain using an Analog-to-Digital Converter (ADC).

### 2.5. Measurement Algorithm

**Figure 2:**
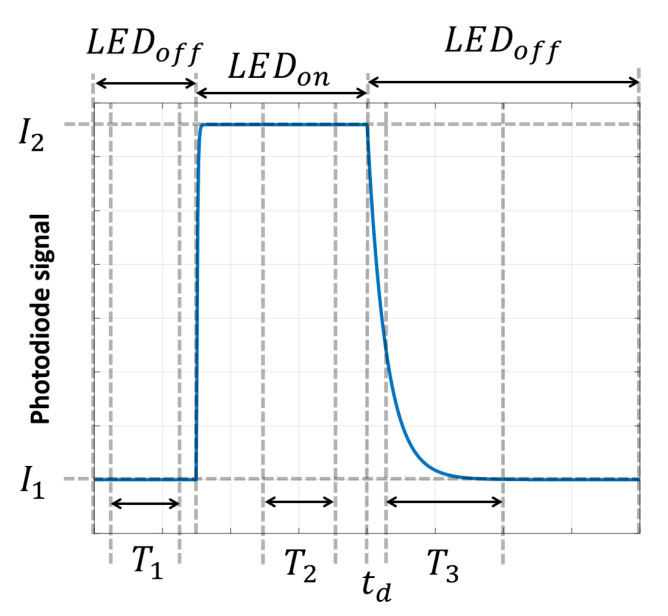
Measurement scheme based on three time windows.

The phosphorescence intensity and lifetime of the luminescence decay were obtained following a method similar to that proposed by López-Ruiz et al. [13], which is based on the integration of the luminescence signal over three different windows of time, as schematized in Figure 2. First, before the optical excitation of the sample, the signal obtained from the photodiode is integrated over the time window $T_{1}$. During this interval the value of the signal is almost constant and corresponds to the offset due to the photodiode dark current. Secondly, the sample is optically excited after which, once the luminescence reaches the steady state, the signal is integrated over a time window $T_{2}$. Finally, the LED is turned off again and, after waiting a delay time $t_{d}$ to avoid the background fluorescence, the signal is integrated again over the time window $T_{3}$, which is wide enough to cover the whole decay time.

Therefore, both the offset ($I_{1}$) and intensity of the luminescence in excited steady state ($I_{2}$) can be obtained from Equation (1).

(1) $$I_{i}=\frac{D_{i}}{T_{i}},\;i=1,\;2$$

where $D_{i}$ is the result of the integration of the signal over the time window $T_{i}$.

Moreover, it has already been demonstrated that for a time window much longer than the lifetime, $T_{3}\gg \tau$, the value of the mean lifetime of an *n^th^*-order exponential can be obtained assuming a mono-exponential decay with notional lifetime τ [20]. Thus, the integration of the decay over the time window $T_{3}$ results in

(2) $$D_{3}=\int_{td}^{T_{3}}(I_{2}e^{-\left(t/\tau \right)}+I_{1})dt≅I_{2}\tau +I_{1}\left(T_{3}-t_{d}\right)$$

provided that $T_{3}\gg \;\tau$ and $t_{d}\ll \;\tau$.

Then, the lifetime can be determined from Equations (1) and (2) as follows:

(3) $$\tau =\frac{D_{3}-\frac{D_{1}}{T_{1}}\left(T_{3}-t_{d}\right)}{\frac{D_{2}}{T_{2}}}$$

Furthermore, we also obtained the ratio of change in the amplitude of the phosphorescence decay in order to calculate the concentration of the analyte. For that, we used the area measured over the time window $T_{3}$, as indicated in Equation (4).

(4) $$\frac{I_{o}}{I_{0}}=\frac{D_{3_{0}}-\frac{D_{1}}{T_{1}}\cdot T_{3}}{D_{3}{}_{i}-\frac{D_{1}}{T_{1}}\cdot T_{3}}$$

where $I_{0}$ is the amplitude in absence of the analyte, and $I_{i}$ this e amplitude obtained for a given analyte concentration.

### 2.6. Room-Temperature Phosphorescence Hb Determination

The typical procedure was carried out as follows: A series of solutions from 0.24 mg·mL^−1^ CDs to 15 mM H_2_O_2_ were prepared and diluted to 3 mL with phosphate buffer (0.02 M NaH_2_PO_4_/Na_2_HPO_4_, pH 4.7) to achieve the final concentration of 2.8 × 10^−3^ mg·mL^−1^ of CDs and 0.5 mM H_2_O_2_. Next, increasing volumes of the 300 μM Hb stock solution to calibrate or Hb containing the sample were added to previous solutions, and the RTP intensities of the solutions were determined after 5 min at room temperature using standard 10 mm quartz cells. The average of the data from three independent measurements were obtained. The same procedure was applied to the preparation of the samples for Hb determination using the portable device.

### 2.7. Real Sample Measurement

Blood samples were obtained from laboratory volunteers; 10 µL of sample were diluted to 7 mL with Milli-Q water and incubated for 30 min to release the Hb from the red blood cells. After centrifugation at 3000 rpm for 10 min, 10 µL of the sample were added to 3 mL of buffer solutions with of 2.8 × 10^−3^ mg·mL^−1^ CDs and 0.5 mM H_2_O_2_. Then, the procedure for Hb determination was applied.

## 3. Results and Discussion

### 3.1. Carbon Dot Characterization

In general, the formation of CDs doped with N mainly involves two processes: condensation and carbonization. During these processes, the carboxyl and hydroxyl groups of citric acid and the amino groups of ethylenediamine undergo complex condensation and carbonization reactions involving intramolecular condensation to form small-molecule fluorophores, such us 5-oxo-1,2,3,5-tetrahydroimidazo[1,2a]pyridine-7-carboxylic acid [29] as well as carbon core-containing nitrogen-doped carbon dots. The resulting molecular fluorophores are hypothesized to be located on the surface and/or inside the CDs.

**Figure 3:**
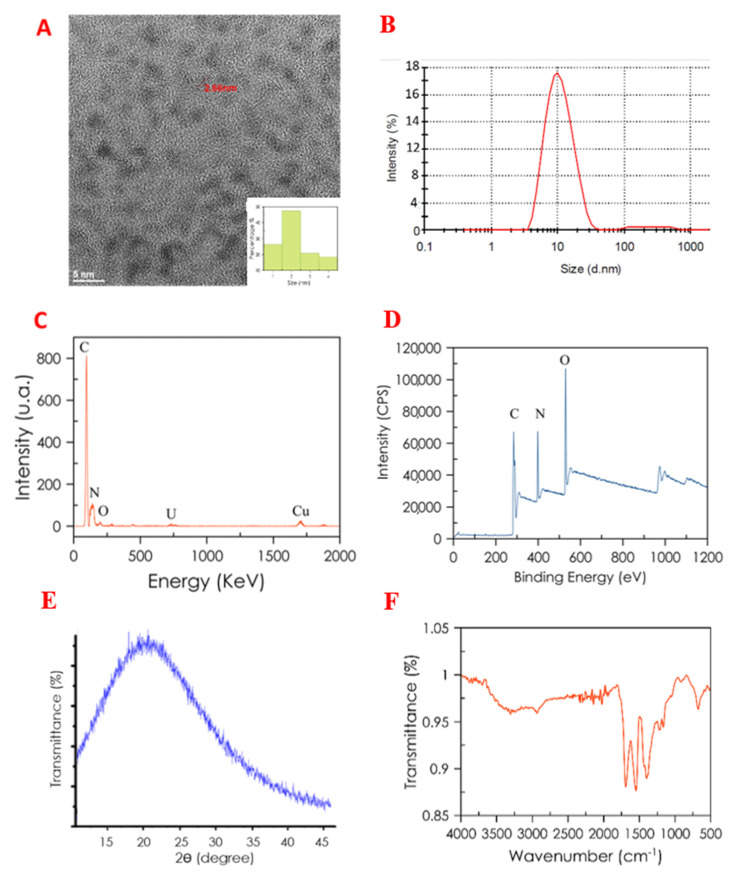
Characterization of carbon dots (CDs). (**A**) HR-TEM image; (**B**) CDs size distribution using DLS; (**C**) EDX spectrum. (**D**) XPS spectra: C1s peak: 284 eV; N1s peak: 398 eV, and O1s peak: 530 eV. (**E**) XRD pattern and (**F**) FTIR spectrum of CDs.

The size, morphology and structure of the synthesized CDs were studied by HR-TEM, EDX, XPS, FTIR, and XRD. The HR-TEM image of the CDs (Figure 3A) shows that the CDs were spherical with a low degree of agglomeration. The particle size distribution histogram was obtained from HR-TEM and presented in Figure 3A. The sizes of CDs were distributed in a narrow range of 1–5 nm with an average particle size of 2.5 nm in HR-TEM, and particle sizes of CDs obtained by DLS measurement (Figure 3B) were shown to be ~10 nm, which is the normal size of CDs. Elemental analyses by EDX were performed to discover the composition of the CDs, showing that C, N, and O atoms were present in the composition of CDs (see Figure 3C). XRD was carried out, as seen in Figure 3E. A diffraction peak was observed at 2Φ  =  21.5°, which is typical for the amorphous crystal phase.

The FTIR spectrum of the CDs (Figure 3F) showed the characteristic bands of COOH stretching at 3440 cm^−1^ and 1637 cm^−1^; NH bending at 1585 cm^−1^; C–N stretching at 1122 cm^−1^; and CH asymmetric and symmetric stretching at 2950 and 2820 cm^−1^, respectively. These data suggest the presence of different functional groups such as –OH, –COOH, and –NH_2_ on the surface of CDs [30].

Additionally, the elemental composition of the CDs was performed by XPS surface analysis. As expected, the obtained data for the elemental composition of the CDs indicate the presence of a carbon peak (C1s) at about 284 eV, an oxygen peak (O1s) at about 530 eV, and a nitrogen peak (N1s) at about 398 eV. Additionally, the atomic quantification shows 69.37% C1s, 16.62% O1s, and 14.02% N1s atoms, (Figure 3D). 

### 3.2. Optical Properties of the Synthesized CDs

**Figure 4:**
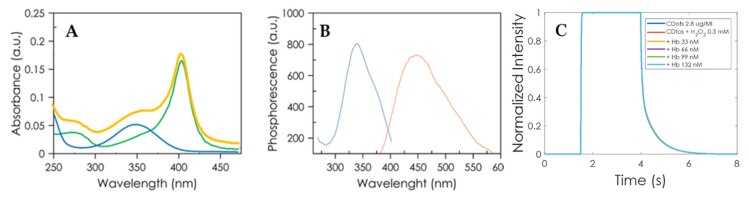
(**A**) UV spectra of CD solution (2.8 × 10^−3^ mg·mL^−1^ CDs, blue line), Hb solution (0.5 µM Hb, green line), and CDs/Hb solutions (yellow line), all in 0.02 M NaH_2_PO_4_/Na_2_HPO_4_ (pH = 4.7) buffer. (**B**) Room temperature phosphorescence spectra of CD solution (2.8 × 10^−3^ mg·mL^−1^ CDs), blue line excitation spectrum and red line emission spectrum. (**C**) Normalized photoluminescence decay for different Hb concentrations. The offset of the curves was removed.

The presence of different surface groups such as hydroxyl, amine, or carboxyl on CDs improves their stability and modulates the luminescent properties, paving the way for new sensing applications. The prepared CDs showed a UV–Vis absorption spectrum with a maximum at 350 nm, attributed to the n–π * transition of C=O bonds [31] (Figure 4A), and an emission spectrum ranging from approximately 400 to 550 nm, with an emission behavior independent of the excitation, and a maximum luminescence wavelength at 442 nm, with a full width at half maximum (FWHM) of around 110 nm (Figure 4B).

It must be taken into account that for the synthesized carbon dots, they exhibited two photoluminescence processes that competed simultaneously (that is, fluorescence and phosphorescence emissions), both emitting near the same spectral range.

Yan et al. [32], in his study of the same CDs studied by us from citric acid and ethylenediamine, attributes the observed phosphorescence to both the aromatic carbonyl groups present and to the graphitic structure of the CDs, which is similar to the aromatic polycyclic structure; these polycyclic aromatic hydrocarbons are a family of compounds well known for their phosphorescent properties.

The relative fluorescence quantum yield (QY) of the CDs was determined using a slope method described in the literature [33]. The relative QY fluorescence of the CDs obtained using quinine sulfate as standard was 0.41 ± 0.05 as the average of three measurements.

### 3.3. The Mechanism of Quenching Carbon Dots Luminescence by Hb

The Hb spectrum presented a significant band at 407 nm and another minor band at 280 nm. The aqueous solution of luminescent CDs upon addition of Hb showed an overlap in the absorption spectra, a quenching, and a small red-shift in their emission spectra. The quenching was attributed to the inner filter effect (IFE) by the partial overlap of the Hb spectra with the CDs emission spectra [34]. To confirm this mechanism, in addition to the overlap in the spectra, we calculated the lifetimes of the system. This was confirmed by the phosphorescence lifetime calculated with the portable instrument (see Figure 4C), in which these decays had a mean lifetime of τ = 228.8 ± 4.5 ms, which was calculated using a time window of *T*_1_ = *T*_2_ = *T*_3_ = 1.5 s and a time delay of *t_d_* = 1 ms. These results show that the developed portable instrument is capable of measuring phosphorescent lifetimes, which is very interesting for future sensing applications.

Moreover, the addition of H_2_O_2_ to the solution containing CDs and Hb dramatically increased the luminescence quenching of CDs. Barati et al. [31] suggest a different quenching mechanism from IFE. In short, in the first step, Hb reacts to H_2_O_2_ generating reactive oxygen species (ROS), mainly hydroxyl ∘OH and superoxide $O_{2}^{\circ -}$ radicals, which occurs with heme group degradation and iron release. The subsequent oxidation of the surface hydroxyls of the CDs modifies the surface structure, leading to luminescent quenching [35].

### 3.4. Assay Optimization

**Figure 5:**
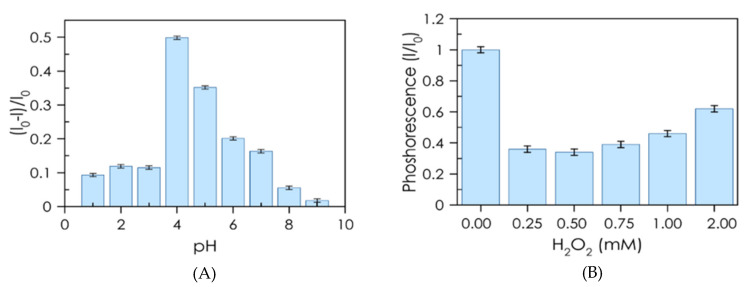
(**A**) pH dependence of luminescence quenching; 2.8 × 10^−3^ mg·mL^−1^ CDs, 0.5 mM H_2_O_2_, 1 µM Hb in 0.02 M phosphate buffers. (**B**) Influence of H_2_O_2_ concentration on luminescence quenching; 2.8 × 10^−3^ mg·mL^−1^ CDs, 0.15 µM Hb in 0.02 M NaH_2_PO_4_/Na_2_HPO_4_ pH = 4.7 buffer.

Firstly, a study of the effect of the solution reaction time, pH, and H_2_O_2_ was conducted. The equilibration time was studied, finding that 6 min is sufficient to obtain stable measurements. The influence of pH on the response was investigated in the range of 1.5–9.5 (0.02 M NaH_2_PO_4_/Na_2_HPO_4_ buffer). As shown in Figure 5A, the greatest attenuation occurred at pH 4, dramatically decreasing in both more acidic and basic media. Likewise, the concentration of H_2_O_2_ exerted a great effect on the attenuation of luminescence (Figure 5B), with 0.5 mM being the optimal value.

### 3.5. Prototype Implementation and App Application

**Figure 6:**
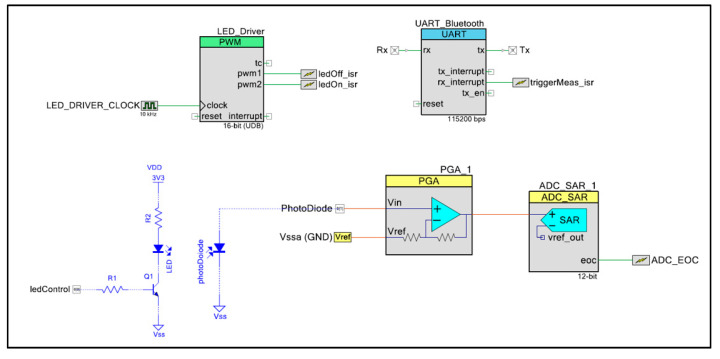
Diagram block of the developed instrument showing the electronics part and the measurement setup.

**Figure 7:**
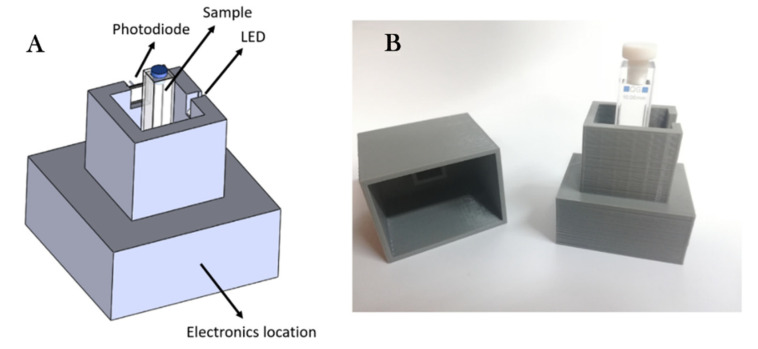
A 3D model (**A**) and real view (**B**) of the final portable instrument.

As described above, the phosphorescence intensity and lifetime measurement algorithm was implemented in a reconfigurable device whose full hardware configuration is shown in Figure 6 and implemented in the portable device in Figure 7. This design was implemented using the PSoC Creator Integrated Design Environment (IDE), which makes it possible to configure the different hardware modules and implement the firmware. As seen in Figure 1, the LED driver responsible for controlling the excitation source was implemented through a pulse-width modulation (PWM) module. This module generates two digital signals, which are connected to two hardware triggered interrupts to turn on/off the LED. This implementation based on interruptions makes it possible to monitor the time intervals in which the excitation LED is turned on/off very accurately. The signal obtained from the photodiode is amplified (*A_v_* = 4 *V*/*V*) using a programmable gain amplifier (PGA) to adapt the signal to the dynamic range of the analog digital converter (ADC) module, which was configured as singled-ended. The ADC converts the signal recorded from the photodiode to the digital domain at a sampling rate of 250 kHz and a resolution of 12 bits, generating an end of conversion (EOC) interruption every time that a new conversion is completed. Therefore, the integration of the signal over each time window yields Equations (5) and (6) as follows:

(5) $$D_{i}=\overset{N=\frac{T_{i}}{Ts}}{\underset{j=0}{\sum }}V_{ADC}{}_{j}\cdot T_{S}=F_{S}\cdot \overset{T_{i}\cdot F_{S}}{\underset{j=0}{\sum }}V_{ADC_{j}},\;i=1,2$$

(6) $$D_{3}=\overset{N=\frac{T_{3}}{Ts}}{\underset{j=0}{\sum }}(V_{ADC_{j}}-\frac{D_{1}}{T_{1}})\cdot T_{S}=F_{S}\cdot \overset{T_{3}\cdot F_{S}}{\underset{j=0}{\sum }}(V_{ADC}{}_{j}-\frac{D_{1}}{T_{1}})$$

where *T_S_* and *F_S_* are the sampling period and the sampling frequency, respectively, and $V_{ADC_{j}}$ is the value of the $j^{\mathrm{th}}$ conversion.

Once the lifetime is calculated using Equation (3), it is sent by the Bluetooth interface, implemented as an external Bluetooth Low-Energy (BLE) module based on the CC2541 system-on-chip (SoC) (Texas Instrument, Dallas, TX, USA). A full-duplex universal asynchronous receiver-transmitter (UART) module is the interface with the BLE module, which works as a slave of a central/master device. In this work, the master device is a smartphone that runs an app for data visualization and triggers new measurements.

### 3.6. Interference Study

**Figure 8:**
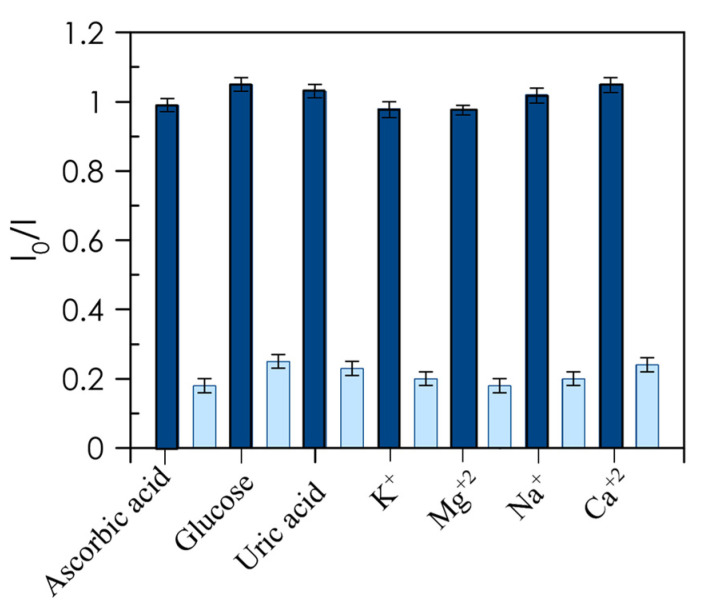
Selectivity of RTP assay. Response of interferents (10 µM for ascorbic acid, glucose, and uric acid and 0.1 mM for cations) and response of Hb (100 nM) in the presence of interferents.

One of the major challenges in the determination of Hb is the selectivity required in the presence of interfering ions and various biologically important species commonly found in real samples that may hamper the analytical application to Hb sensing. To evaluate whether this approach is highly specific for Hb, the quenching of CD suspensions was recorded in the presence of different interfering species, both molecules and ions (final concentration of 10 µM) typically found in blood samples, i.e., ascorbic acid, glucose, uric acid, K^+^, Mg^2+^, Na^+^, and Ca^2+^. None of these species elicited a discernible effect on the phosphorescence response of CDs/H_2_O_2_ for the determination of Hb (Figure 8). These results confirm that neither inorganic nor organic analytes found in blood interfere with our assay, validating the selectivity of the phosphorescence system towards Hb.

### 3.7. Analytical Characterization of the Portable Luminescent Instrumentation

The feasibility of the developed instrument was tested using the CDs as a phosphorescent probe and measuring the change in quenching phosphorescence in the presence of Hb at different assay concentrations.

**Figure 9:**
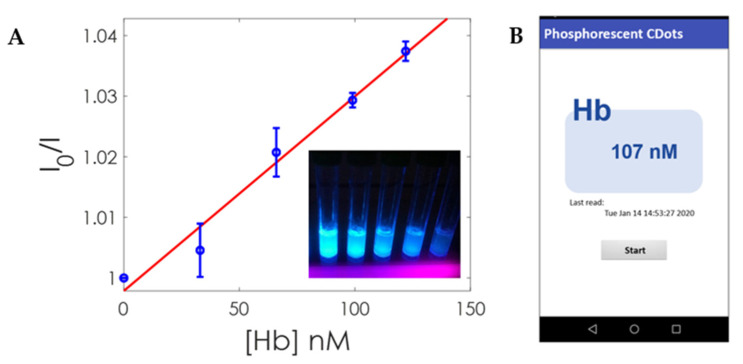
(**A**) Intensity based (*I*_0_/*I*) Stern–Volmer plot for different concentrations of Hb. Inset shows a UV-illuminated photograph of CDs with different concentrations of Hb. (**B**) App screenshot showing the result of one measurement.

Furthermore, we extracted the intensity-based ($I_{0}/I$) Stern–Volmer plot using Equation (4) for the different Hb concentrations. This plot makes it possible to define a calibration function to determine the concentration of analyte (Hb) from the ratio between the intensity in the absence of Hb and the intensity measured for a certain concentration of Hb. The calibration function obtained in this case is indicated in Equation (7), a linear dynamic range from 19 nM to 125 nM and a correlation coefficient of 0.9976. The LOD was calculated using the standard criteria, namely LOD = 3σ/slope, where σ is the standard deviation (*n* = 20) of the difference in luminescence intensity between a CD solution and a blank solution; in this case, the criterion for the quantification limit (LOQ) was 10σ/slope. With this criterion, the value of the LOD was 6.2 nM, and the LOQ was 18.8 nM. Then, the Hb concentration could be extracted using this calibration curve and the ratio of intensities previously measured. Finally, this value was sent by the Bluetooth interface to the Android app (Figure 9). The app, in addition to simply working as a display of this value, is also used to trigger new measurements. A comparative study of different analytical performance for colorimetric and fluorimetric detection of GSH is presented in Table 1.

(7) $$\frac{I_{0}}{I}=320\cdot 10^{-6}\cdot \mathrm{Hb}+1;\;R^{2}=0.9976$$

### 3.8. Application to Real Sample Analysis

Despite the good selectivity of phosphorescence CDs to detect Hb in the presence of interfering ions, the problem of unspecific interferences is hard to tackle due to the inherent compositional complexity of real biological samples. To validate the selectivity of the proposed assay for Hb determination in blood, samples from two healthy human volunteers were analyzed after a large dilution of samples to reduce any interference. Moreover, the quantification after addition of a known amount of Hb (0.040 and 0.080 µM) in the diluted blood samples showed a good recovery percentage (Table 2). These results demonstrated the good accuracy of phosphorescence CDs for Hb determination in human blood.

## 4. Conclusions

The determination of Hb was carried out using a portable reconfigurable device developed in our laboratory for the room-temperature phosphorescence measurement of CDs. The development sensor combines the nanoparticles with the RTP detection, resulting in a significant improvement in the selectivity and sensitivity of the detection of Hb.

The portable device allowed for the determination of Hb with good sensitivity, and a detection limit of 6.2 nM for Hb was reached within a linear range up to 120 nM in concentration. The analytical applicability of the portable instrument was successfully demonstrated by blood analysis. The adjustment of the measuring range using a reconfigurable electronic system has great potential for future applications. This instrument offers the advantages of versatility, portability and accuracy for RTP measurements.

## Figures and Tables

**Table 1:** Comparison of performance of proposed method for Hb with literature.

Method	Materials	Linear Range (nM)	LOD (nM)	References
Colorimetry	Curcumin nanoparticles	15.5–620	1.55	[16]
Colorimetry	G-quadrupolex DNAzymes	1–120	0.64	[36]
Fluorimetry	Molecular Imprinting Polymers	25–3000	7.8	[21]
Fluorimetry	Terbium complexes	9–540	3	[37]
Fluorimetry	CdHgSe QDs	4–440	2	[38]
Fluorimetry	CDs	1–4000	0.12	[39]
Fluorimetry	BSA-AuNCs	1–250	0.36	[17]
Fluorimetry	AuNCs	10–2000	5	[22]
Fluorimetry	Silicon nanoparticles	50–4000	40.0	[40]
RTP	CDs	19–125	6.2	This work

**Table 2:** Hb determination in real blood samples.

Samples	Concentration of Hb				Recovery %
	Detected (µM)	Blood (mM)	Added (µM)	Recovered (µM)	
Volunteer 1	0.050	10.500	0.040	0.052	93.6
			0.080	0.085	105.9
Volunteer 2	0.045	9.450	0.040	0.038	95.0
			0.080	0.087	108.4
